# Associations between number of siblings, birth order, eating rate and adiposity in children and adults

**DOI:** 10.1111/cob.12438

**Published:** 2021-01-12

**Authors:** Christina Potter, E. Leigh Gibson, Danielle Ferriday, Rebecca L. Griggs, Christle Coxon, Margot Crossman, Ray Norbury, Peter J. Rogers, Jeffrey M. Brunstrom

**Affiliations:** ^1^ Nutrition and Behaviour Unit, School of Psychological Science University of Bristol Bristol UK; ^2^ Department of Psychology University of Roehampton London UK; ^3^ College of Health, Medicine and Life Sciences, Division of Psychology Brunel University London Uxbridge UK; ^4^ National Institute for Health Research Bristol Biomedical Research Centre University Hospitals Bristol NHS Foundation Trust and University of Bristol Bristol UK

**Keywords:** adults, birth order, BMI, children, eating rate, number of siblings

## Abstract

Eating quickly is associated with eating larger amounts at mealtimes and faster eaters tend to have a higher BMI. Evidence suggests that sibling structure influences the development of childhood eating behaviours. We hypothesized that number of siblings and birth order might play a role in the development of eating rate. In two UK studies, children in Bristol (*n* = 132; Study 1) and adults and children in London (adults *n* = 552, children *n* = 256; Study 2) reported their eating rate, number of siblings, and birth order. A BMI measurement was obtained and in Study 2 waist circumference was recorded. Ordered logistic regression was used to examine effects of sibling structure on eating rate and linear regression assessed effects of eating rate on BMI. Faster eating was associated with higher BMI and a larger waist, in children and adults (ps < .01). In Study 1, first‐born children were twice as likely to eat faster compared to children who were not first‐born (*P* < .04). In Study 2, only‐child adults reported eating slower than adults who were not first‐born (*P* < .003). Additionally, higher number of siblings was associated with faster eating rate in children from Bristol (*P* < .05), but not in children from London. London adults without siblings ate slower than those with two or more (*P* = .01), but having one sibling was associated with eating faster than having two or more (*P* = .01). These findings reveal how birth order and number of siblings might influence eating rate. Exploring these relationships through direct observation would be beneficial in future studies.

**What is already known about this subject**

- Currently, one in five children in the UK lives with obesity.
- Family structure, including sibling number and birth order, can contribute to the development of unhealthy eating behaviours in childhood. Many eating behaviours learned in childhood, including eating rate, are risk factors for obesity in children.
- Faster eating rate is associated with increased risk of obesity. Foods that are eaten quickly tend to be consumed in larger amounts, and when meal size is fixed, eating at a faster rate reduces satiation.

**What this study adds**

- Results from both studies are consistent with previous research showing a positive relationship faster eating rate and BMI.
- Children with siblings were more likely to have a faster eating rate. There was no clear relationship between birth order and eating rate, though there was some evidence that first‐born children were more likely to have a faster eating rate than children without siblings.

## 1 INTRODUCTION

Foods that are eaten quickly tend to be consumed in larger amounts,^1^, ^2^ and when meal size is fixed, eating at a faster rate reduces satiation.^3^ Further, eating quickly is positively associated with body mass index (BMI) in adults^4^ and children.^5^ There is evidence that eating rate is modifiable and that reducing eating rate can lead to weight loss. One study found that training adolescents with obesity to moderate their speed of eating leads to a sustained reduction in BMI.^6^ A number of studies have manipulated basic properties of foods, finding that factors such as physical^7^ and textural^8^, ^9^, ^10^ characteristics can slow eating rate and increase satiation. Understanding the development of eating behaviours that might promote overeating and lead to obesity, particularly in children, is essential. Childhood obesity is a serious problem in many countries, with obesity being prevalent in one of every five children in the United States and in England, according to the Center for Disease Control and National Child Measurement Programme for England.^11^, ^12^ Further, there is evidence that obesity promoting behaviours learned in childhood may track into adulthood.^13^

Family structure plays an important role in the development of eating behaviours in children. There is evidence of an association between parents' perceptions of their children's eating preferences and child BMI. One study found that, regardless of children's own preferences, parents who believed their child would like to consume or was capable of consuming larger food portions were more likely to have a child with obesity.^14^ Children in this study reported similar preferred and maximum portion sizes, regardless of their BMI, suggesting that parents might provide larger portions to children if they themselves believe their child would like to eat more. It is unclear whether the same relationship exists between parents' perceptions of their children's eating speed. That is, if a parent believes their child is a fast eater it is possible that they will promote this behaviour in their child.

In addition to parental influence, siblings also play an important role in the early development of obesity‐promoting eating behaviours. According to the most recent UK census data, of the nearly 6.5 million families in the UK with dependent children, 2.5 million those families have two or more children.^15^ Several systematic reviews have examined the role of sibling structure on the development of obesity in children. Evidence suggests that children without siblings (only children) are more likely to be overweight or have obesity than children with siblings and, for children with siblings, those with fewer siblings are more at risk of developing unhealthy eating habits (eg, increased portion size and snack food consumption). Regarding birth order, youngest siblings seem to be more at risk of becoming overweight or have obesity than their older siblings. Importantly, these associations were found to persist over time..^16^, ^17^

Although previously unexplored, there are several reasons to suspect that sibling number might shape eating rate. First, children tend to eat at a faster rate when dining with others^18^ and they consume more food than when eating alone.^19^, ^20^ Since many children eat with their siblings, those with siblings might develop a tendency to eat at a faster rate. Second, absolute group size may play a role. In one study, children consumed 30% more food when eating in a large group (nine children) compared to when eating in a smaller group (five children).^18^ Therefore, children with more siblings may have greater propensity for increased eating rate. Additionally, siblings are not only familiar with one another (as is also the case with friends) but they may also share food resources at mealtimes which may introduce an element of competition. In the context of eating behaviour, we reasoned that competition might promote an increase in eating rate that is proportionate to the number of siblings in a family. Further, competition may also depend on birth order, with greater competition in older children if caregiver efforts are directed toward younger siblings.

Here, we report results from two studies that explored the influence of number of siblings and relative birth order on eating rate, both in children and in adults. We predicted that greater sibling number and higher birth order (older children) would be positively associated with eating rate. In Study 1, these relationships were assessed in children. In Study 2, we sought to replicate and extend this work by assessing eating rate in a second cohort of children, and we recruited adults with a broad range of BMI to determine whether effects of sibling number and birth order are associated with differences in adiposity in this cohort.

## 2 STUDY 1: METHODS

### 2.1 Participants

Participants (*N* = 132) were English‐speaking 5‐ to 11‐year‐old children. They were recruited along with one or more parents (or primary caregiver) at an interactive science center in Bristol. The study protocol was approved through the University of Bristol's Faculty of Science Human Research Ethics Committee. No form of remuneration was offered.

### 2.2 Eating rate measurement

Parents were asked to report their child's eating rate by selecting one of three options: (a) slow, (b) average, and (c) fast in response to the question “*How would you describe your child's typical eating speed?*” This simple response format has been previously validated against direct observations of eating rate.^21^

### 2.3 Birth order and number of siblings

Parents were also asked to report their child's number of siblings and order of birth relative to those siblings. Birth order was subsequently coded as either “only child”, “first born”, or “not‐first‐born” (ie, middle and youngest children).

### 2.4 Anthropometric measures

Height was measured to the nearest 0.1 cm using a portable stadiometer and weight was measured to the nearest 0.1 kg using a Tanita TBF‐531 scale. Parents reported their child's date of birth. Using this information, child body mass index (BMI) was converted to SD scores (BMI SDS) adjusted for age and sex (LMS Growth v2.77^22^).

### 2.5 Procedure

Data from Study 1 were collected alongside a larger study of parent and child interactions around food.^14^ Parents read an information sheet before providing written consent for their child to take part. Working with a researcher to verbally complete a questionnaire, parents provided information about their child's eating rate, birth order, and number of siblings. Parents were asked to include in this account their child's biological siblings as well as any other dependent children currently living with their child. A measure of the child's height and weight was then recorded. Finally, the parent was offered a copy of the consent form and a debriefing sheet. Each testing session lasted approximately 5 minutes.

### 2.6 Statistical analyses

Separate ordinal logistic regressions were used to test the hypotheses that number of siblings (only child, one sibling, or two or more siblings) and birth order (only child, first born child, or not‐first born child) are related to eating rate. To control for potential developmental differences in eating rate, child age was included in each regression model. The effect of eating rate on child BMI SDS was tested by linear regression. Statistical analyses were performed using SPSS version 23.0.0.2 (SPSS Inc., Chicago, Illinois).

## 3 STUDY 1: RESULTS

### 3.1 Demographics

Children (*N* = 132) had a mean age of 7.6 (SD: 1.74) years and the majority (*n* = 98) were lean (BMI SDS < 1.04,^23^). Their age, gender, BMI, number of siblings, birth order, and eating rate are shown in Table 1.

**TABLE 1:** Participant characteristics in Study 1

	Descriptives^a^	%
Age (years)	7.6 (1.7)	‐
Gender (*female*)	71	53.8
BMI SDS	0.48 (0.97)	‐
Number of siblings		
Only child	21	15.9
One	82	62.1
Two or more	29	22.0
Birth order		
Only child	21	15.9
First born	63	47.7
Not‐first born	48	36.4
Eating rate		
Slow	56	42.4
Average	53	40.2
Fast	23	17.4

^a^ Values for age and BMI SDS are displayed as means (SD). All other values are displayed as frequencies and percentages (*n* = 132).

### 3.2 Number of siblings and eating rate

There was a significant relationship between number of siblings and eating rate, explaining about 9% of the variance (Table 2: Nagelkerke pseudo *R* ^2^ = .092). Compared to children with two or more siblings, children without siblings (only children) report a significantly slower eating rate (Table 2 *: P* = .028). Children with one sibling tended to eat more slowly than those with two or more siblings (ie, about half as likely to eat as fast), although not significantly so (*P* = .095). In this model, child age was not significantly related to eating rate (Table 2).

**TABLE 2:** Relationships between sibling number, birth order and eating rate in Study 1

	Eating rate		
	Odds ratio (95% CI)	*p*	Model fit
Number of siblings			χ^2^ (3, *n* = 132) = 11.08, *p* = .011
Age	1.21 (0.99 to 1.46)	.056	
Only child	0.28 (0.09 to 0.87)	.028	
One sibling	0.50 (0.22 to 0.89)	.095	
Two + siblings	1^a^	‐	
Birth order			χ^2^ (3, *n* = 132) = 12.50, *P* = .006
Age	1.19 (0.97 to 1.43)	.088	
Only child	0.74 (0.27 to 2.05)	.569	
First born	2.16 (1.04 to 4.57)	.040	
Not‐first born	1^a^	‐	

^a^ the reference category in the ordinal logistic regression.

### 3.3 Birth order and eating rate

There was a significant relationship between birth order and eating rate, explaining about 10% of the variance (Table 2: Nagelkerke pseudo *R* ^2^ = .103). Compared to non‐first‐born children, first‐born children were about twice as likely to have a faster eating rate (*P* = .040). There was no significant difference between the reported eating rate of non‐first‐born children and children without siblings (*P* = .569). Child age was not a significant factor (*P* = .088) in this model (Table 2).

### 3.4 Eating rate and BMI SDS

There was no observed relationship between eating rate and BMI SDS in this study (*F*_2,131_ = 0.149, *P* = .862, *β* = −0.052).

## 4 INTERIM DISCUSSION

This study is the first to provide evidence for an association between number of siblings, birth order, and eating rate. It was found that having siblings is associated with faster eating rate; however, this was only reliably evident when comparing children without siblings to those with two or more siblings. Additionally, results indicate that first‐born children eat at a faster rate than non‐first‐born (ie, youngest and middle) children do. One potential concern is that the association between number of siblings and eating rate observed in Study 1 is confounded by child age. Previously, it was found that older children eat at a faster rate than younger children (0.45 bites/min faster, 95% CI: 0.12‐0.79).^24^ This is likely due to developmental differences as children get older (ie, the ability to feed themselves increases with age, and nutritional demands are greater). However, the observed relationship between eating rate and child age in this model, while supporting that finding, did not reach significance.

Previous research has demonstrated that having fewer than two siblings leads to a greater risk of obesity in children^25^, ^26^ and adolescents.^27^ Curiously, results from Study 1 indicate that having fewer than two siblings was associated with slower eating rate, which is typically thought to be associated with lower food intake^2^ and lower BMI.^4^ By contrast, the finding that older siblings have a faster eating rate (which may promote overconsumption) complements earlier research demonstrating that first‐born children are at higher risk of obesity than their younger siblings.^28^ The sample from Study 1 was predominantly lean and this may explain the lack of association found between eating rate and BMI SDS. Therefore, a primary aim of Study 2 was to assess the effects of eating rate on adiposity, via associations with BMI and also waist circumference; the latter being a more reliable index of associations between adiposity and health outcomes.^29^, ^30^

Additionally, Study 2 aimed to replicate the effects from Study 1 and to extend these findings to both adults and children. A potential concern from Study 1 was that the 3‐point response scale for eating rate limited the variability observed in responses. Moreover, measures of the eating rate of children were obtained from their parents. Therefore, in Study 2 all participants reported their own eating rate, with an expanded 5‐point response scale. As with many other eating behaviours, eating rate develops in childhood^31^ and may sustain through to adulthood. Although adults likely no longer live with their siblings, the effects of sibling structure on eating rate in their childhood may still be evident in adulthood. Therefore, in Study 2, exploring the relationship between eating rate, sibling structure, and BMI was a primary objective in both children and adults.

## 5 STUDY 2: METHODS

### 5.1 Participants

Adults (18‐85 years old; *n* = 317 women, 235 men) and children (7‐17 years old; *n* = 144 girls, 112 boys) were recruited from visitors to the Science Museum in London, UK, who volunteered to take part in a “Live Science” public engagement event. The study protocol was approved through the local University of Roehampton Human Research Ethics Committee. Monetary compensation was not provided. Children under seven were not permitted to take part, in accordance with Science Museum ethical requirements.

### 5.2 Eating rate measurement

Participants provided a self‐report of their eating rate with the following response options: (a) very slow, (b) relatively slow, (c) medium, (d) relatively fast, and (e) very fast.

### 5.3 Birth order and number of siblings

As in Study 1, participants reported their number of siblings (only child, one sibling, or two or more siblings) and their birth order (only child, first‐born, or non‐first‐born).

### 5.4 Anthropometric measures

Participants' height (to the nearest mm; portable stadiometer; no shoes), weight (to the nearest 0.1 kg; light clothing) and waist circumference (Seca waist tape at rib to iliac crest midpoint) were measured under supervision from the researchers on‐site in London. Adult BMI was calculated as m/kg^2^. For children, BMI was converted to BMI SD scores (BMI SDS) adjusted for age and sex (LMS Growth v2.77^22^).

### 5.5 Procedure

Participants read an information sheet before providing written consent (or assent) to take part; children were consented by a parent or guardian. All participants (adults and children) provided their own self‐reported eating rate, birth order, and number of siblings, followed by a measure of their height and weight. Participants completed the questionnaires for themselves and data on the relationships between participants (eg, if an adult was a parent to a child participant) was not collected. Finally, participants received a debriefing sheet, which detailed further aims of the research.

### 5.6 Statistical analyses

As in Study 1, separate ordinal logistic regressions were used to test effects of number of siblings (only child, one sibling, or 2 or more siblings) and birth order (only, first‐born, or non‐first‐born) on eating rate. Separate linear regressions were used to test effect of eating rate on child BMI SDS and adult BMI. The effect of eating rate on child and adult waist circumference was tested by multiple linear regression adjusting for age. Separate multiple regression models were used to explore the relationship between number of siblings, birth order and age for both children and adults in Study 2. Statistical analyses were performed using SPSS version 23.0.0.2 (SPSS Inc., Chicago, Illinois).

## 6 STUDY 2: RESULTS

### 6.1 Demographics

Children had a mean age of 10.5 (SD: 2.67) years. Adults had a mean age of 31.7 (SD: 11.9) years. Further participant demographics regarding age, gender, BMI, number of siblings, birth order, and eating rate can be found in Table 3.

**TABLE 3:** Participant characteristics in Study 2

	Children		Adults	
	Descriptives^a^	(%)	Descriptives^a^	(%)
Age	10.5 (2.7)	‐	31.7 (11.9)	‐
Gender *(female)*	141	54.4	317	57.4
BMI	18.42 (3.12)	‐	25.6 (5.1)	‐
BMI SDS	0.42 (1.04)	‐	‐	‐
Waist circumference (cm)	63.9 (9.3)		83.6 (13.2)	
Number of siblings				
Only child	44	17.4	58	10.4
One	121	47.8	235	42.0
Two or more	88	34.8	266	47.6
Birth Order				
Only child	95	37.5	58	10.4
First‐born	41	16.2	207	37.0
Not‐first‐born	73	28.9	294	52.6
Eating rate				
Very slow	26	10.3	16	2.9
Relatively slow	55	21.7	73	13.1
Medium	107	42.3	181	32.5
Relatively fast	48	19.0	229	41.1
Very fast	17	6.7	58	10.4

^a^ Values for age, BMI, and BMI SDS are displayed as means (SD). All other values are displayed as frequencies and percentages. For children *N* = 259 and for adults *N* = 559.

### 6.2 Number of siblings and eating rate

In children, there was no association between number of siblings and eating rate (Table 4). Although the model was significant (*P* < .001), this was driven by child age and not sibling number.

**TABLE 4:** Effects of number of siblings and birth order on eating rate in children and adults in Study 2

	Eating rate	*P*	Model fit
	Odds ratio (95% CI)		
Children			
Number of siblings			χ^2^ (3, *n* = 253) = 18.29, *p* < .001
Age	1.20 (1.09 to 3.35)	<.001	
Only child	1.72 (0.88 to 3.35)	.111	
One sibling	1.48 (0.79 to 2.80)	.223	
Two + siblings	1^a^	‐	
Birth order			χ^2^ (3, *n* = 253) = 20.54, *P* < .001
Age	1.17 (1.07 to 1.28)	<.001	
Only child	0.56 (0.28 to 1.09)	.091	
First‐born	1.39 (0.84 to 2.29)	.202	
Not‐first‐born	1^a^	‐	
Adults			
Number of siblings			χ^2^ (3, *n* = 559) = 18.35, *P* < .001
Age	0.99 (0.99 to 1.01)	.890	
Only child	0.50 (0.30 to 0.85)	.011	
One sibling	1.55 (1.12 to 2.16)	.010	
Two + siblings	1^a^	‐	
Birth order			χ^2^ (3, *n* = 559) = 13.25, *P* = .004
Age	0.99 (0.98 to 1.01)	.641	
Only child	0.45 (0.27 to 0.76)	.003	
First‐born	1.23 (0.89 to 1.72)	.210	
Not‐first‐born	1^a^	‐	

^a^ the reference category in the ordinal logistic regression.

However, there was a significant relationship between number of siblings and eating rate in the adult sample, explaining about 4% of the variance (Table 4: Nagelkerke pseudo *R* ^2^ = .036). Compared to adults with two or more siblings, adults without siblings (only children) reported a significantly slower eating rate (ie, were half as likely to eat as fast, *P* = .011), whereas adults with one sibling were 55% more likely to eat at a faster eating rate (*P =* .01). There was no relationship between age and eating rate in the adult sample (Table 4).

### 6.3 Birth order and eating rate

As in Study 1, there was mixed evidence for a relationship between birth order and eating rate. In children, while the model was significant and explained about 9% of the variance (Table 4: Nagelkerke pseudo *R* ^2^ = .086), this was again driven by child age (*P* < .001), although there was a tendency for only children to be less likely to eat as fast as those who were not first born (Table 4 *: P = .091*). In adults, compared to adults who were not first born, those without siblings (only children) were significantly less likely to eat as fast (Table 4 *: P* = .003). However, first‐born adults were likely to eat at the same rate as those who were not first born.

### 6.4 Eating rate, BMI and waist circumference

In Study 2, faster eating rate was significantly associated with a higher BMI SDS in children (*F*_1,248_ = 9.01, *P* = .003; 3.5% variance explained) and a higher BMI in adults (*F*_1,553_ = 4.26, *P* = .039; 0.8% variance explained). Results from the age‐adjusted ordinal regression models for children and adults are presented in Table 5: where waist circumference is used for adiposity, adjusting for age disconfounds the increase in waist due to growth in children, as well as a developmental trend for older children and adolescents to eat faster.^24^ In adults, age adjustment disconfounds other influences on waist that are associated with age, such as reduced metabolic rate, hormone sensitivity and physical activity.^32^

**TABLE 5:** Parameter estimates from hierarchical multiple linear regression models of associations between eating rate and waist circumference adjusted for age (Study 2)

Dependent variable	Independent variable	Adjusted *R* ^*2*^	*R* ^2^ change	B	SE	*P*
Child waist	Eating rate	0.40	0.026	1.49	0.45	<.001
Adult waist	Eating rate	0.09	0.012	1.55	0.57	.007

**FIGURE 1:**
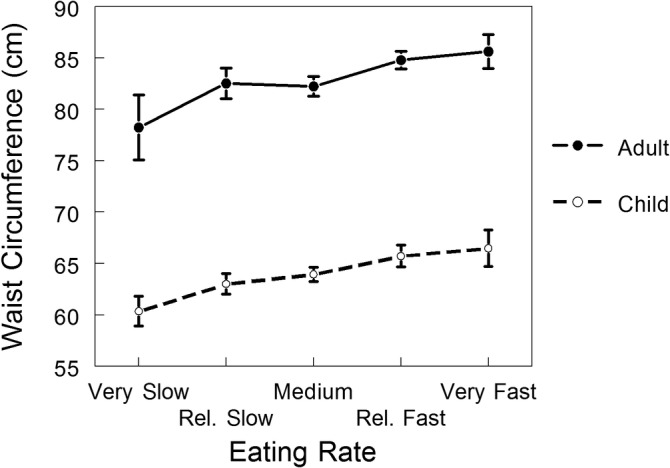
Relationships between eating rate and waist circumference (mean ± SE, adjusted for age) for adults and children (Study 2)

When waist circumference was the outcome, adjusted for age, significant associations between eating rate and child adiposity (*F*_2,247_ = 84.1, *P* < .001; 2.6% additional variance explained) and adult adiposity (*F*_2,543_ = 28.0, *P* < .001; 1.2% additional variance explained) were seen (F‐change *P* = .001, .007, respectively for children and adults). Thus, faster eating rate was modestly but significantly linearly associated with larger waist circumference in children (including adolescents) and adults; these associations are illustrated in Figure 1.

### 6.5 Exploratory analysis: Sibling number, birth order, and BMI

An exploratory research question resulting from these findings is whether number of siblings and birth order relate to child or adult BMI. Separate multiple regression models were used to explore the relationship between number of siblings, birth order and age for both children and adults in Study 2. For children, the model exploring association between number of siblings, birth order and age with BMI SDS was not significant (*P* = .244). For adults, the model exploring these relationships was significant (*P* < .001); however the effect was driven by participant age (*P* < .001) and not by number of siblings (*P* = .760) or birth order (*P* = .847).

## 7 DISCUSSION

Together these findings support the hypothesis that having siblings promotes faster eating rate. However, this evidence was mixed and only evident in the Bristol children and the London adults. Eating rate in the Bristol children was provided by parent report whereas London children and adults provided a self‐report so it may be the case that London children were not accurate in determining their own eating rate. If their parents had been asked to report eating rate for their children, a different pattern of results may have been observed. Importantly, parents in Study 1 reported their children's eating speed rather than children providing a self‐report and so the results must be interpreted with caution.

There was mixed evidence in support of a relationship between birth order and eating rate across the two studies. In Study 1, non‐first‐born children ate more quickly than only children did and this same pattern was evident in adults in Study 2. Interestingly, the relationship between birth order and eating rate was not evident in children in Study 2. Indeed, for children in Study 2 the primary driver of eating rate was child age. It may be the case that some children in the same household do not eat meals together and so future studies exploring self‐reported eating rate should include a measure of how many people are present during mealtimes. Further, it may be the case that adults' current living situation (ie, living alone vs living with others) also impacts their eating rate, which limits the extent to which effects of birth order can be observed. Unfortunately, current living situation was not assessed in the adults in Study 2 and, with this information, it might be possible to isolate the independent effects of birth order and household number on eating rate. Notably, these studies found non‐significant relationships directly between sibling number and birth order with BMI. This is perhaps because the differences in eating rate are too small to drive a sufficient increase in energy intake and BMI. Alternatively, perhaps the effect of eating rate is counteracted by other unmeasured factors, such as food availability, where there may be less food available but still eaten at a faster rate.

Our results also support previous studies showing a relationship between body mass and eating rate,^33^, ^34^, ^35^, ^36^ and in Study 2 this association was observed in both adults and children. This relationship was not observed in Study 1, likely to due to the predominantly lean cohort of children. Nevetherless, on its own, eating rate accounted for only a small proportion of variance (less than 4%) in both BMI and waist circumference. To date, many studies have found an association between eating quickly and increased energy intake (for a recent systematic review, see Robinson et al.^2^). However, few intervention studies have been conducted with the aim to reduce eating rate to combat obesity. One randomized control trial found that training adolescents with obesity to eat more slowly lead to successful reductions in body weight.^6^ Furthermore, after 12 months of treatment in adolescents, this method (Mandometer) of feedback on eating rate also resulted in reduced blood levels of the appetite hormone, ghrelin, and increased levels of the satiety hormone peptide tyrosine‐tyrosine (PYY), following ingestion of a glucose load.^37^ Thus, training children to eat more slowly may also enhance their responsiveness to their internal satiety cues^38^ rather than external environmental cues (eg, amount of food left on the plate, amount of food available for the group) or purely hedonic aspects of food.^39^

Reducing distraction at mealtimes may also reduce eating rate and increase responsiveness to internal satiety cues. As mentioned previously, eating with others or in the presence of distraction promotes increased energy intake.^40^ Increased number of siblings may promote faster eating due to increased distraction (eg, talking to siblings) and decreased attention to internal satiety cues. In addition, the potentially competitive nature of eating with siblings and its effect on eating rate may differ from adult group eating, where the main effect seems to be to increase meal duration and size, but not eating rate.^41^ Reducing other distractions (eg, television on during meals), may also reduce eating rate and increase sensitivity to satiety cues. Indeed, it has been found that watching a computer game during meals reduces perceived post‐prandial satiety^42^ and may lead to overeating.^43^ Interventions aimed at promoting within‐meal awareness to both parents and children may help to reduce eating rate and subsequent overconsumption. The development of a programmable vibrating fork to regulate eating rate is innovative although as yet unproven for weight control.^44^Foods that are eaten quickly also tend to be selected and consumed in larger portions.^1^, ^45^ Additionally, there is an inverse association between energy density and eating rate, whereby energy‐dense foods tend to be consumed quickly.^1^ When eating with their siblings, children may be more likely to consume larger quantities of high‐energy‐dense foods. Indeed, previous research has found that the consumption of high‐energy‐dense foods (cakes, chocolate) increased by 50% when eating with friends compared to eating alone.^19^ The combined preference for energy dense foods, large portions, and fast eating rate will likely promote overconsumption and obesity. In adults with obesity, sustained interventions targeting eating rate may be necessary to overcome habitual influences on eating of such hedonically attractive foods.^46^

There is a large body of literature exploring the important influence that siblings have on each other during the important developmental years of childhood and adolescence.^47^ However, more research is needed to understand how sibling influences specifically shape obesity‐promoting eating behaviours. Indeed, others have noted the need for more direct examination of the processes through which siblings influence family dynamics.^48^ A future study could explore sibling influences on eating rate through direct examination and measurement, rather than parent estimation or child self‐report. Given that a preference for faster eating appears to develop in childhood and may be preserved through adulthood, family interventions aimed at understanding the development of eating rate should be given priority, as they may help to determine how best to retrain this behaviour at an earlier age.

## CONFLICT OF INTEREST

The authors declare no conflict of interest.
